# *Drosophila* HUWE1 Ubiquitin Ligase Regulates Endoreplication and Antagonizes JNK Signaling During Salivary Gland Development

**DOI:** 10.3390/cells7100151

**Published:** 2018-09-26

**Authors:** Yifat Yanku, Eliya Bitman-Lotan, Yaniv Zohar, Estee Kurant, Norman Zilke, Martin Eilers, Amir Orian

**Affiliations:** 1 Rappaport Research Faculty of Medicine, Technion-Israel Institute of Technology, Haifa 31096, Israel; yifaty@technion.ac.il (Y.Y.); eliyabit@technion.ac.il (E.B.-L.); 2 Institute of Pathology, RAMBAM Medical Center, Haifa 30196, Israel; yanivz75@gmail.com; 3 Faculty of Natural Sciences, University of Haifa, Haifa 3498838, Israel; ekurant@univ.haifa.ac.il; 4 Genome-Scale Biology Research Program Institute of Biomedicine University of Helsinki, 00290 Helsinki, Finland; norman.zielke@helsinki.fi; 5 Theodor Boveri Institute, Biocenter, University of Würzburg, D-97074 Würzburg, Germany; martin.eilers@biozentrum.uni-wuerzburg.de

**Keywords:** HECT, HUWE1, ubiquitin, salivary gland, endoreplication, JNK, dMyc, dmP53

## Abstract

The HECT-type ubiquitin ligase HECT, UBA and WWE Domain Containing 1, (HUWE1) regulates key cancer-related pathways, including the Myc oncogene. It affects cell proliferation, stress and immune signaling, mitochondria homeostasis, and cell death. HUWE1 is evolutionarily conserved from *Caenorhabditis elegance* to *Drosophila* *melanogaster* and Humans. Here, we report that the *Drosophila* ortholog, d*HUWE1* (CG8184), is an essential gene whose loss results in embryonic lethality and whose tissue-specific disruption establishes its regulatory role in larval salivary gland development. dHUWE1 is essential for endoreplication of salivary gland cells and its knockdown results in the inability of these cells to replicate DNA. Remarkably, dHUWE1 is a survival factor that prevents premature activation of JNK signaling, thus preventing the disintegration of the salivary gland, which occurs physiologically during pupal stages. This function of dHUWE1 is general, as its inhibitory effect is observed also during eye development and at the organismal level. Epistatic studies revealed that the loss of *dHUWE1* is compensated by dMyc proeitn expression or the loss of dmP53. dHUWE1 is therefore a conserved survival factor that regulates organ formation during *Drosophila* development.

## 1. Introduction

HECT (E6-AP carboxyl terminus)-type E3 ubiquitin ligases regulate diverse cellular processes during development as well as in adult metazoan tissues [1,2,3,4]. One highly evolutionary conserved HECT ubiquitin ligase is HUWE1 (known also as HectH9, MULE, LASU1, ARF-BP1) [5,6,7]. Human HUWE1, a large ubiquitin ligase with a molecular weight of 482 kDa, is composed of 4374 amino acids. At its C-terminus region, it harbors the conserved HECT domain, which is characteristic of HECT ubiquitin ligases [8,9]. Substrate recognition by HUWE1 is likely mediated by other domains outside the catalytic HECT domain. A BH3 domain located within its N-terminal region mediates its interaction with Mcl-1 and adjacent UBA and WWE motifs are required for binding to K63-linked polyubiquitin chains. Specifically, upon immune activation by IL-1, HUWE1 generates K48-branched chains on substrates already characterized by existing K63-linked chains previously catalyzed by TRAF6, culminating in mixed-K48/63ubiquitin branched chains. These branched chains likely protect these substrates from de-ubiquitination and are required for NF-κB signal transduction amplification [10].

Among the direct substrates of HUWE1 are regulators of DNA synthesis and cell cycle progression, the response to DNA damage, mitochondria homeostasis, cell proliferation, cell death, and NF-κB and JNK signaling pathways [10,11,12,13,14,15]. In cancer, HUWE1 targets tumor suppressor proteins, such as the checkpoint protein TopBP1 and the apoptosis-related protein Mcl1, for proteasomal degradation [16,17]. The oncogenic or suppressive functions of HUWE1 in cancer are context dependent, as HUWE1 ubiquitinates and enhances c-Myc transcriptional activity, which promotes tumorigenesis [5,18]. It also, however, targets N-Myc for degradation during neurogenesis in mice [19], and has tumor suppressive activities in colon cancer where Wnt signaling is hyperactivated [20,21].

HUWE1 is critical for normal development and organogenesis, as it is required for the degradation of the Atoh transcription factor and affects axonal branching during neurogenesis. Likewise, its *C. elegans* ortholog, *ELL*, is required for neuronal development [22,23,24,25]. In vertebrates, HUWE1 is required for myogenesis. It ubiquitinates both MyoD and TBP, inhibiting the differentiation of myoblasts and affecting promoter selection by the basal transcription machinery [26,27]. In accordance with its role in neurogenesis, mutations in the HUWE1 gene have been associated with familial cases of the Brooks-Wisniewski-Brown and Juberg-Marsidi (JMS) X-linked intellectual disability (XLID) syndromes [28]. Less is known, however, about the developmental role(s) of HUWE1 in non-neuronal tissues in vivo.

Here, we report that the fly ortholog of HUWE1, *dHUWE1* (CG8184), is an essential gene in *D. melanogaster*, and is required for salivary gland development and endoreplication. dHUWE1 also prevents premature activation of the JNK pathway, thereby regulating the timing of salivary gland disintegration. Remarkably, the phenotypes associated with loss of *dHUWE1* are suppressed by either loss of *dmP53* or the expression of dMyc.

## 2. Materials and Methods

### 2.1. Fly Strains and Genetics

Flies were maintained on yeast/cornmeal/molasses/malt extract medium at 25 °C or at 29 °C, where indicated. Alleles used in this study: UAS-Reaper was a gift from Eli Arama. UAS-Hid, UAS-Eiger, and *puc^69^*::lacZ, were a gift from Adi Salzberg, UAS-dMyc was as described [29]. The following lines were from the Bloomington Stock Center: UAS-dHUWE1 (BSN#15528), UAS-Hep (BSN #6406), UAS-Dcr-2.D1 (BSN #24650), UAS-dTRAF6 (CG10961, BSN#58991). The following RNAi lines were obtained from VDRC: dHUWE1 RNAi: (v26935), dMyc RNAi (v2947), dmP53 RNAi (v26351), Grnd RNAi (v43454), TRAF6 RNAi (v110266) (v16125), Tab2 RNAi (100326). The following Gal4 lines were used: w1118 *hey*-Gal4;+;+ [30]. *ptc*-Gal4, (BSN #2017) *GMR*-Gal4 (BSN #1104), *C253*-GAL4 (BSN #7011), *en*-Gal4, UAS-GFP/Cyo, and ey-GAL4 were from Adi Salzberg. The RGB cell cycle tracer UAS-RGB=P{w{+mC} = UAS-nlsCdt1N101EBFP2-T2A-nlsCycBN96-nlsCycBN285tdTomato-T2A EGFPPCNA} II.1 was a kind gift from Christian Lehner [31].

### 2.2. Antibody Production

Polyclonal mouse antiserum against dHUWE1 was generated by immunizing BALB/c BYJ Rb(8.12) 5BNR/J mice (Jackson Labs) with a GST-fusion to dHUWE1 fragments (amino acids 4158-4208). Western blotting and immunofluorescence of salivary gland and embryonic tissues were performed to test antibody specificity (Supplementary Figure S1, and not shown).

### 2.3. Other Antibodies Used

α-dMyc mAb (P4C4B10, 1:10; [32]); mouse polyclonal α-Fibrillarin (1:2000, [33]) Rabbit α-Nop60b at 1:250 (immunostaining) and 1:1000 (Western blot) were a gift from S. Poole. Rabbit α-β-Gal (1:500 #55976) and mouse α-Actin (1:4000) were purchased from MP Biomedicals. Secondary antibodies used: Alexa Fluor^®^ 488 goat anti-mouse IgG (H+L); Alexa Fluor^®^ 568 goat anti-mouse IgG (H+L); Alexa Fluor^®^ 568 goat anti-rabbit IgG (H+L); DNA dyes used: Draq5 (1:5000 889-001-R200, Biostatus) and DAPI (1:1000 D9542-1MG, Sigma).

### 2.4. Salivary Gland and Fat Body Dissections, Tissue Preparation, Confocal Microscopy and IMARIS Analysis

*Drosophila* tissues were dissected from the indicated third instar wandering larvae, collected, and transferred to cold PBS solution for dissection. Larvae were cut, and dissected tissues were subsequently transferred to an Eppendorf tube containing 500 μL fixation solution (4% formaldehyde 0.1% Triton X-100 in PBS). Tissues were fixed at RT for 20 min, washed thoroughly with 100% methanol three times followed by three washes with ethanol, and processed for indirect immunohistochemistry. Immunofluorescence and confocal microscopy were performed as previously described [29]. In brief, 100μL fixed tissues were washed with PBS, and 0.1% Triton X-100 (PBX) to remove ethanol traces and transferred to a blocking solution for 60 min (PBST; PBS, 0.1% Triton X-100, 2% BSA, 2% Goat Serum). Tissues were incubated overnight at 4 °C with the indicated primary antibody diluted in PBST. Next, tissues were washed thoroughly with PBX×4 for 15 min each. Secondary antibody was then added along with DAPI/DRAQ5 and the tissues were incubated in the dark at RT for 2 h followed by washes with 4× PBX and 2× PBS. Tissues were then mounted on slides for imaging using Zeiss LSM 700 laser confocal microscope. Data analysis was performed using IMARIS software for data visualization (Bitplane).

### 2.5. EdU Labeling

EdU live staining of salivary glands was performed in 250 μL of 2× EdU working solution (Click-iT EdU imaging kit Invitrogen C10338) with 250 μL added Ringer’s solution, and incubated at RT on a nutating mixer for 60 min. Salivary glands were then fixed with 4% Para formaldehyde for 30 min at RT, and subsequently stained with the EdU reaction cocktail for 30 min (Click-iT EdU imaging kit).

### 2.6. Terminal Deoxynucleotidyl Transferase dUTP Nick End Labeling (TUNEL) Assay

Third instar larvae were dissected in cold PBS and salivary glands were fixed in freshly prepared 2% para-formaldehyde for 60 min at RT. Subsequently, the glands were washed with PBS×2 for 5 min and re-suspended in permeabilization solution for 2 min. Next, tissues were washed with PBS and re-suspended in the labeling solution and labeled using a cell death detection kit, TMR red, Roche #12156792910). Finally, tissues were washed thoroughly with PBS, stained with DAPI and mounted on slides for signal detection by confocal microscopy.

### 2.7. Plasmids and Constructs for Expression in S2 Cells

UAS-attB-dHUWE1-short (a.a. 4140-5146), was cloned into the UAS attB vector using standard PCR cloning techniques.

### 2.8. RNAi and Measurement of Protein Stability in S2 Cells

*Drosophila* Schneider S2 cells were maintained using Schneider’s media (Invitrogen, Carlsbad, CA, USA) and supplemented with 10% FBS and 10 mM glutamine at 25 °C. dsRNA molecules for RNAi targeting of either dHUWE1 or GFP (control) were prepared and delivered to S2 cells using the MegaScript RNAi Kit (Ambion, Austin, TX, USA) and similar to reference [29]. Plasmid transfection was performed using FugeneHD® reagent. Dynamic cyclohexamide chase experiment was performed as described in reference [34]. In brief, 3 × 10^6^ cells were incubated with 10 μg/mL cycloheximide (Sigma #01810) for the indicated time and washed twice with PBS×1. Cell extracts were prepared in 100 μL RIPA buffer supplemented with a protease inhibitors cocktail (Roche #11873580) and 120 μg of cell extract was resolved on SDS-PAGE. Protein levels were determined by Western blot analysis with the indicated antibody.

## 3. Results

**Figure 1:**
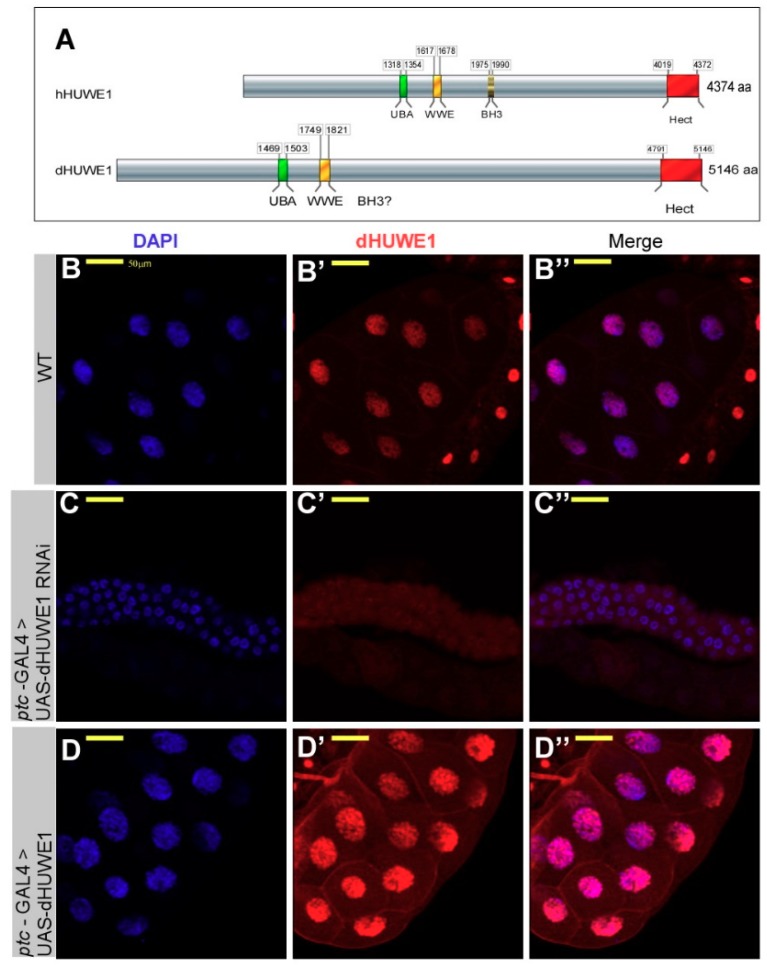
dHUWE1 is required for salivary gland development. (**A**) Schematic diagram of human and *Drosophila* dHUWE1 proteins (not to scale). UBA (green) and WWE (yellow) are ubiquitin-binding motifs. BH3; Bcl2-homology domain, and HECT domain is the catalytic core (red). (**B**–**D”**) Indirect immunofluorescence confocal microscopy images of wild-type and the indicated transgenic lines stained with anti-dHUWE1 antibody (red). DAPI (blue) marks DNA, and the scale bar is 50 μm. (**B**) Endogenous levels of dHUWE1. (**C**–**D”**) Expression of UAS-dHUWE1-RNAi (**C**,**C”**) or UAS-dHUWE1 (**D**,**D**”) under the control of *ptc*-Gal4.

Using *Drosophila* as a model system, we characterized functional aspects of dHUWE1 (CG8184), the fly ortholog of HUWE1. Located on the X chromosome, it encodes a 5146 amino acid protein. Although the fly orthologue is highly homologous to HUWE1, it lacks a definitive BH3 domain (Figure 1A). Interestingly, some of its key substrates and regulators, including ARF and Mcl proteins (which interact with the BH3 domain of HUWE1), are absent in *Drosophila*. Thus, *Drosophila* represents a simplified and ancient regulatory gene network that contains key cancer-related genes like dMyc and *dm*P53. Using a self-generated polyclonal antibody to dHUWE1, we observed that dHUWE1 protein is ubiquitously expressed in the embryo and in larval tissues including the salivary gland and wing discs (Figure 1, Supplementary Figure S1, and see Materials and Methods). A p-element insertion line within the dHUWE1 gene resulted in lethality, and no homozygous females or hemizygous male adult were recovered, suggesting that dHUWE1 is an essential gene. We therefore decided to focus on the role of dHUWE1 in a specific tissue, the developing salivary gland.

### 3.1. dHUWE1 Regulates Salivary Gland Development and Endoreplication

In *Drosophila*, dMyc plays an important role in regulating cell growth and cell size [32,35,36]. Specifically, in salivary gland cells, dMyc positively regulates endoreplication, a process during which sequential DNA-synthesis cycles without cell divisions give rise to polyploid nuclei (~1350 C) [37,38,39].

Since Myc proteins are key substrates of HUWE1 in vertebrates, we began by characterizing the expression pattern and effects of modulating dHUWE1 expression in the third instar larvae salivary gland. As Figure 1B–B” shows, endogenous dHUWE1 protein is highly expressed in the nuclei of salivary gland cells. Next, we tested the impact of dHUWE1 over-expression on salivary gland morphology using an inserted p-element containing a UAS-activating sequence located 500 pb upstream to the transcription start site. This UAS insertion enables the conditional expression of the full-length endogenous dHUWE1 protein under the control of specific GAL4 lines [40]. We also used a dHUWE1-specifc UAS-transgenic RNAi (VDRC #26935) to deplete dHUWE1 (Supplementary Figure S2). We used either *ptc*-Gal4 or *hey*-Gal4 to direct the expression of these transgenic lines to the salivary gland cells [41], which gave similar results.

We observed that RNAi-mediated depletion of *dHUWE1* resulted in the absence of a dHUWE1 immunostaining signal as well as in small, underdeveloped salivary glands that contain roughly the same number of cells as do wild-type salivary glands (100–120 cells/gland). These cells were, however, characterized by small nuclei (Figure 1C–C”). Over-expression of dHUWE1 did not result in notable changes at the organ level. However, the nuclei of salivary gland cells were slightly larger than of wild-type cells (Figure 1D–D”, see Discussion). In line with these results, we found that *dHUWE1*^+/−^ heterozygous females are characterized by a reduced size of salivary gland (Figure S5E) and so we conclude that dHUWE1 is required for salivary gland development.

**Figure 2:**
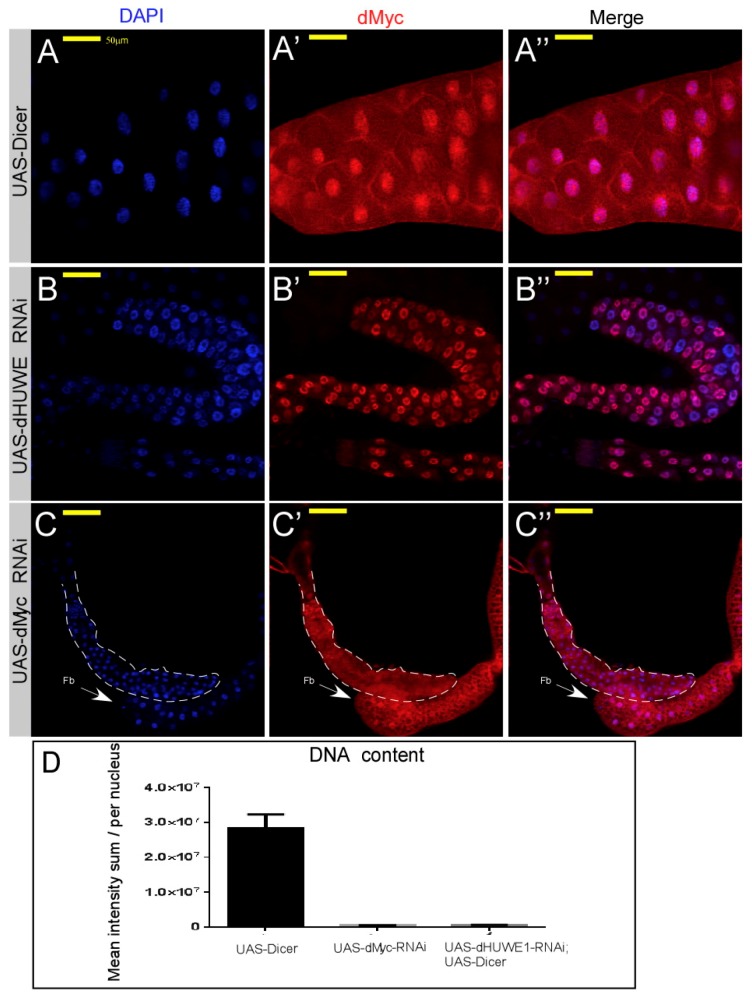
Loss of dHUWE1 results in a small salivary gland that expresses dMyc protein. (**A–C”**) Indirect immunofluorescence confocal microscopy images of wild-type (**A**–**A”**) and the indicated UAS-RNAi transgenic lines (dHUWE1, **B**–**B”**; dMyc, **C**–**C”**) immuno-stained with anti-dMyc antibody (red). DAPI (blue) marks DNA, and the scale bar is 50 μm. The dotted line in **C**–**C”** indicates the edges of the small salivary gland. Fb; fat body tissue. (**D**) Indirect quantification of the DNA content of salivary gland cells derived from the indicated transgenic lines using Imaris technology® (*n* = 10 *p* < 0.001).

We further determined the levels of dMyc protein in dHUWE1-depeleted salivary glands using a dMyc antibody (B10 mAb) [29,32]. We detected a significant signal for dMyc protein in dHUWE1-targeted salivary glands compared with wild-type glands (compare Figure 2A–B”). The overall shape of the gland was, however, small, similar to dMyc-targeted glands (Figure 2C–C”). Both dHUWE1 as well as dMyc knockdown cells exhibited reduced endoreplication and reduced DNA content as indirectly measured by Imaris software (Figure 2D) [41]. This observation fits well with a previous FACS analysis of dHUWE1 RNAi as well as dMyc RNAi knockdown in *Drosophila* S2 which resulted in an increase in G1, and reduced cell size [42].

**Figure 3:**
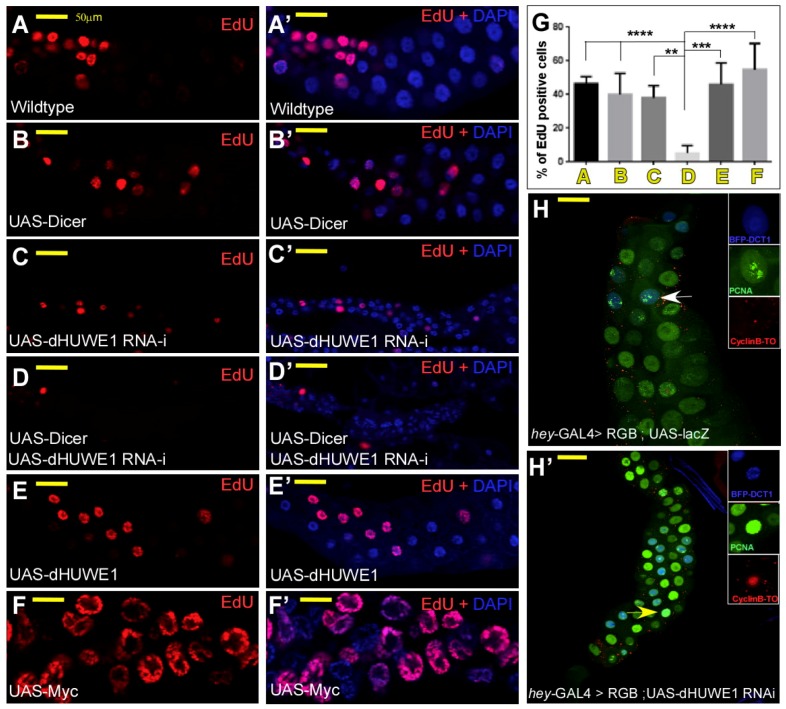
RNAi-mediated depletion of dHUWE1 results in failure to enter S-phase and aberrant cell cycle phasing. (**A–F’**) Representative indirect immunofluorescence confocal microscopy images of wild-type or the indicated transgenic lines under the control of *ptc*-Gal4 labeled with EdU, indicating active DNA synthesis. DAPI (blue) marks DNA and the scale bar is 50 μm. (**G**) Quantification of **A**–**F’** ** p < 0.01; *** p < 0.001; **** p < 0.0001). (**H,H’**) Cell-cycle phasing of control (**H**), and dHUWE1-RNAi (**H’**) targeted salivary gland cells using the in vivo transgenic RGB tracer (see text and methods for details). Arrows indicate cells shown in the inset.

Reduced ploidy can originate from two different scenarios [30,43]: (1) A defective mitotic-to-endocycle switch or (2) impaired endoreplication. The first scenario results in extremely small shoe-string-like salivary glands that are devoid of EdU/BrdU staining [44]. Although ploidy levels were severely reduced, the dHUWE1-depleted salivary glands likely underwent a limited number of endoreplication cycles. To test this notion directly, we assessed DNA synthesis in dHUWE1-depeleted salivary glands by monitoring EdU incorporation. In contrast to wildtype or UAS-Dicer expressing cells, reducing dHUWE1 levels resulted in a significant reduction in EdU-positive cells (Figure 3A–D’, and quantified in 3G), indicating that it is dHUWE1 loss that impairs the endoreplication machinery rather than a failure in the mitotic-to-endocycle switch. Consistent with previous studies that indicated that increased dMyc expression promotes endoreplication in *Drosophila* salivary glands [35], we found increased ploidy levels and EdU-positive cells upon ectopic expression of dMyc. Overexpression of dHUWE1 resulted, however, in an EdU pattern that was highly similar to wild-type control (Figure 3E–F’, and quantified in 3G), implying that ectopic dHUWE1 cannot accelerate the rate of endoreplication in a manner similar to dMyc.

We further analyzed the cell-cycle phasing of control and HUWE1 cells using the FUCCI-type cell cycle tracer RGB [31]. FUCCI technology [45,46] relies on the sequential degradation of fluorescently labeled fragments of cell-cycle regulators such as Cdt1, Geminin or Cyclin B. The RGB cell-cycle tracer consists of a multi-cistronic construct that includes BFP-Cdt11-101 (blue), nuclear-targeted CycB1-96-CycB1-285 fused to Tomato (CycB-To, red) and EGFP-PCNA (green), separated by T2A auto-cleavage sites [31]. The CyclinB-based probe is targeted by the APC/C-Fzr complex which is active during the endocycle G phase [44], while the Cdt1-based probe is targeted by the S phase-specific CRL4-Cdt2 E3-ligase [47]. The RGB cell-cycle tracker also contains a PCNA-GFP fusion protein, which indicates the onset of S-phase by the appearance of characteristic speckles [48]. In endoreplicating wild-type salivary glands and when the molecular oscillator that drives the endocycle is correctly regulated, the expression of BFP-Cdt11-101 and of NLS- CycB1-96-CycB1-285-Tomato is mutually exclusive [41]. After completing the developmentally preset number of endoreplication cycles, the activity of CRL4-Cdt2 activity ceases, while the activity APC/C-Fzr is upregulated in all nuclei, resulting in salivary glands that only express BFP-Cdt11-101 (Figure 3H). Remarkably, in dHUWE1-depleted salivary glands cells, we observed co-expression of BFP-Cdt11-101 and NLS-CycB1-96-CycB1-285-Tomato (Figure 3H’), suggesting that depletion of dHUWE1 affects the molecular oscillator that drives the endoreplication cycles and eventually causes its collapse and reduced ploidy.

dMyc is required for endoreplication, and in both flies and vertebrates, HUWE1 was shown to selectively enhance the transcription of Myc-activated genes [5,18,49]. We therefore tested whether bona fide dMyc-activated targets are expressed in dHUWE1-targeted salivary gland [50]. We observed that both Fibrillarin and Nop60B proteins are well expressed in targeted cells, (Supplementary Figure S3A–L). Likewise, targeting dHUWE1 in the fat body using the *C253*-GAL4 driver resulted in small underdeveloped fat body cells, yet the expression of Fibrillarin was unaffected (Supplementary Figure S3F–G”). Remarkably, double knockdown of both dMyc and dHUWE1 in salivary gland cells resulted in small cells that exhibit reduced expression of Nop60B (Supplementary Figure S3M–O). Thus, suggesting that Nop60b expression is dependent on dMyc, but not on dHUWE1.

Complementing the above genetic studies, Western blot analysis of S2 cells with reduced dHUWE1 levels revealed protein levels of both proteins that were indistinguishable from corresponding levels in control cells (Supplementary Figure S3Q,R). Thus, loss of dHUWE1 did not impair dMyc protein levels (Supplementary Figure S3P), or the expression of direct dMyc genes tested. However, it resulted in a failure of salivary gland cells to endoreplicate and in the inability of the gland to develop properly.

### 3.2. dHUWE1 Antagonizes JNK Signaling in vivo

**Figure 4:**
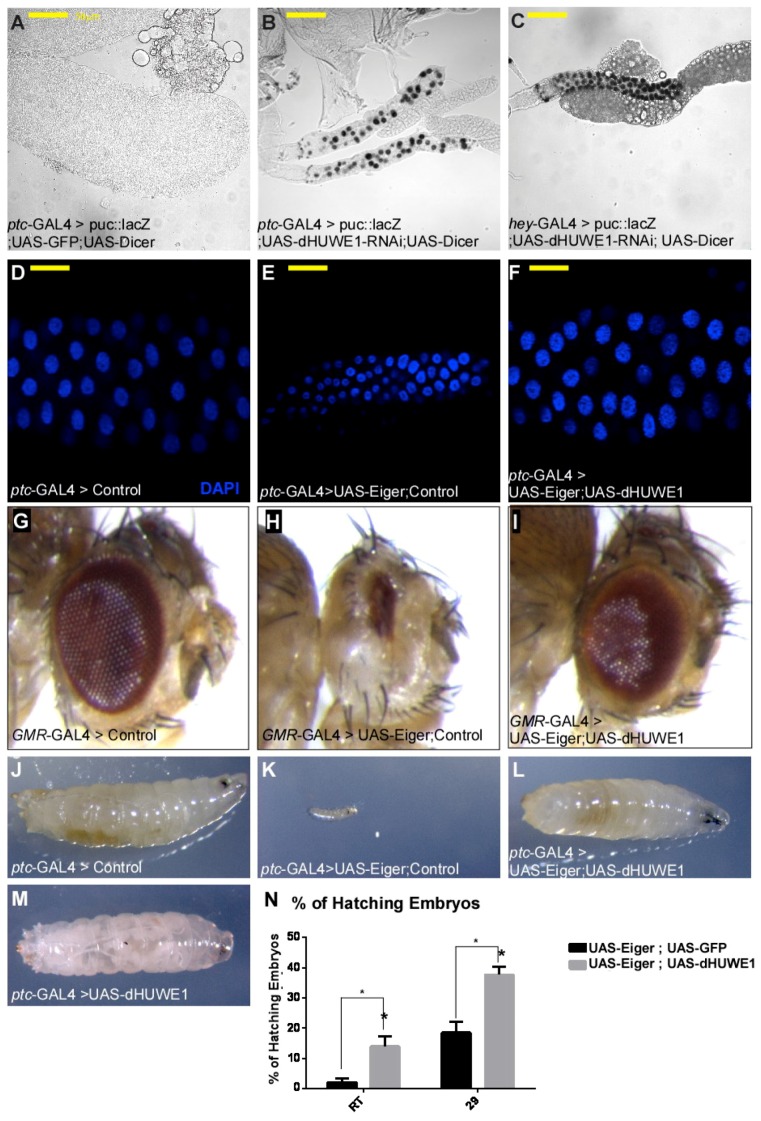
dHUWE1 inhibits JNK signaling and antagonizes the ligand Eiger. (**A–C**) Activation of the JNK pathway *puc^69^*::LacZ-reporter (puc::LacZ, black staining) in control (**A**), or UAS dHUWE1-RNAi under the control of *ptc*-Gal4 (**B**) or *hey*-Gal4 (**C**). (**D–N**) dHUWE1 antagonizes Eiger. Expression of UAS-Eiger results in a small salivary gland (**D** compared to **E**), and small eyes (**G** compared to **H**). Co-expression of UAS-Eiger with UAS-dHUWE1 suppresses these phenotypes (**F**,**I**). (**D**–**F**) Indirect immunofluorescence confocal microscopy images of salivary glands derived from wild-type or the indicated transgenic lines. DAPI marks DNA and scale bar is 50 μm. (**J**–**N**) Expression of Eiger results in lethality during early larval stages that is partially restored by co-expression of UAS-dHUWE1. (**J**–**M**) Representative larval images of the indicated genotypes. (**N**) Quantification of embryonic hatching at indicated temperatures. Expression of UAS-Eiger together with UAS-GFP (control) is shown in black bars, and expression of UAS-Eiger together with UAS-dHUWE1 is shown in gray bars *n* = 1000 *p* = 0.0487 (29 °C, 29); *p* = 0.0426 (Room temperature, RT).

In addition to its cell growth regulatory role, dMyc is also required to prevent JNK pathway activation during retinal glial cell development [51]. In salivary glands, physiological JNK activation takes place during pupal stages and is part of the disintegration process of the gland [52,53]. We therefore tested JNK pathway activation in dHUWE1-targeted salivary glands. We found that loss of dHUWE1 resulted in ectopic activation of the canonical JNK pathway reporter *puc^69^*::LacZ, suggesting that dHUWE1 safeguards against premature JNK activation (Figure 4A–C). In *Drosophila*, activation of the JNK pathway is mediated by the TNFα ortholog, Eiger, and expression of Eiger in salivary gland cells results in small, underdeveloped tissue (Figure 4D,E). Co-overexpression of Eiger together with dHUWE1 in salivary glands restored gland morphology (Figure 4F). The ability of dHUWE1 to antagonize Eiger was also observed in the developing eye and co-expression of UAS-Eiger together with UAS-GFP (control) resulted in a small eye (Figure 4G,H). Remarkably, co-expression of Eiger with UAS-dHUWE1 suppressed Eiger over-expression phenotype and restored eye morphology to that of wild-type (Figure 4G–I). On the organismal level, expression of Eiger resulted in early larval lethality and inability of the developing embryos to hatch; indeed, no adult survivors were observed. Upon dHUWE1 co-expression, however, numerous third instar larvae were observed and 50% of those larvae turned into pupae (Figure 4J–N). In contrast, expression of dHUWE1 alone had no notable effect at the organismal level on the tissue tested, and it did not result in activation of the JNK pathway (Figure 4M, and Supplementary Figure S4E, S5H, S5I). Thus, dHUWE1 antagonizes Eiger-induced JNK activation in the developing salivary gland and in the eye, as well as at the organismal level.

Eiger induces cell death by both caspase-dependent or independent pathways [54]. In *Drosophila*, the caspase-dependent pathway is mediated by the death-inducing genes Hid, Reaper, and Grim. When expressed in the developing eye, these genes induce cell death, resulting in small adult eyes. Unlike the ability of dHUWE1 to inhibit Eiger-induced activation of the JNK pathway, co-expression of dHUWE1 failed to suppress the small eye phenotype induced by Hid or Reaper expression (Supplementary Figure S4A–E). Moreover, dHUWE1 inactivation resulted in TUNEL positive signal, an indication for early events of cells death (Supplementary Figure S4F–H’). Thus, suggesting that dHUWE1 impinges on the non-caspase dependent branch of the JNK-regulated cell death pathway.

To identify potential target(s) for dHUWE1 within the JNK pathway we first tested whether dHUWE1 suppress the small eye phenotype observed upon expression of Hemipetrous (Hep), a downstream component of the JNK pathway. Co-expression of dHUWE1 was unable to suppress the small eye phenotype observed upon expression of Hep (Supplementary Figure S5A–D). Thus, suggesting that the substrate of dHUWE1 is likely situated upstream to Hep. Therefore, we tested whether reduction of upstream component of JNK pathway will suppress the small salivary gland phenotype observed in *dHUWE1*^p^ heterozygous mutant females. Using this assay, we observed that RNAi-mediated knockdown of the Eiger receptor Grindelwald (Grnd), or the ubiquitin ligase dTRAF6, and to a lesser extent the kinase TAB partially compensated dHUWE1 heterozygosity (Supplementary Figure S5E). As expected, expression of dTRAF6 in salivary glands resulted in underdeveloped and small salivary glands with small cells, similarly to the observed upon the expression of Eiger, or the knockdown of dHUWE1 (Supplementary Figure S5F,G). Taken together, these results suggest that the substrate(s) of dHUWE1 is genetically situated at the level of the dTRAF6/TAB signaling complex.

### 3.3. dHUWE1 Genetically Interacts with dMyc and dmP53

**Figure 5:**
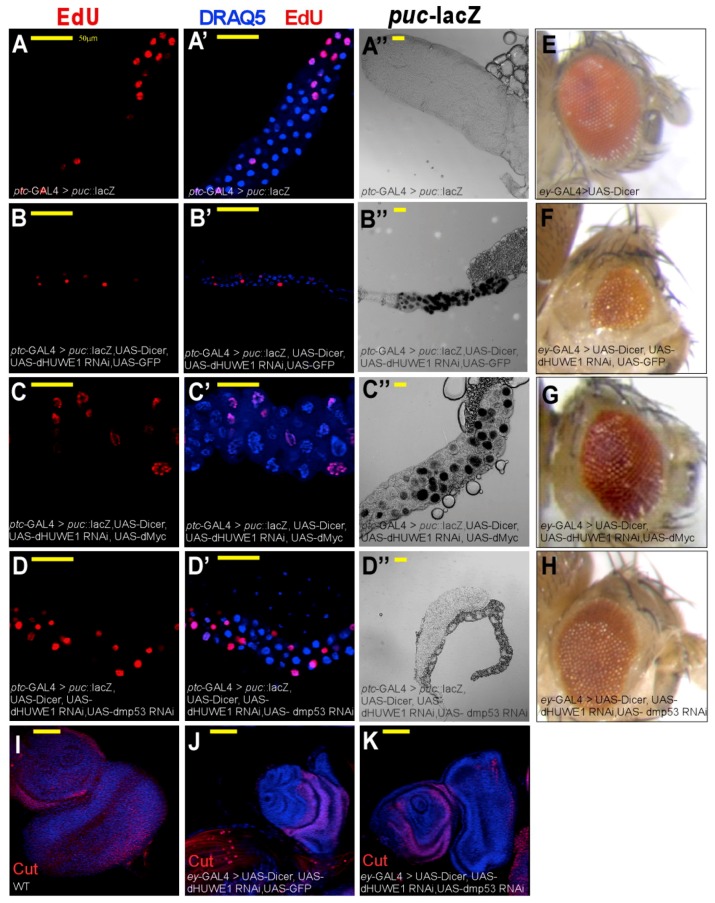
Eliminating *dm*P53 or expression of dMyc suppresses phenotypes associated with loss of dHUWE1. (**A–D’**) Indirect immunofluorescence confocal microscopy images of salivary glands derived from wild-type and the indicated transgenic lines under the control of *Ptc*-Gal4 labeled with EdU to indicate active DNA synthesis. DAPI (blue) marks DNA and the scale bar is 50 μm. (**A”–D”**) Expression of the JNK pathway reporter *puc^69^*:: LacZ (black) in salivary glands derived from control and indicated transgenic lines under the control of *Ptc*-Gal4. (**E–H**) Adult eye morphology in wild-type (**E**), and the indicated UAS-transgenic lines (**E**–**H**), under the control of the eye-specific Gal4, *eyeless*-Gal4 (*ey*-Gal4). (**I**–**K**) Confocal images of eye imaginal discs of wildtype (**I**) or the indicated transgenic lines (**J**,**K**) under the control of *ey*-Gal4 immuno stained with anti-Cut antibody (red) and DAPI that marks DNA.

Both Myc and P53 are intimately linked to HUWE1 and, in *Drosophila*, regulate endoreplication and the JNK pathway. We therefore preformed epistatic studies between *dHUWE1* and *dMyc* or *dmP53*. Specifically, dmP53 is required for JNK activation, an activation that is delayed in a *dmp53* mutant background [55]. We found that loss of *dmP53* resulted in suppression of the phenotype observed in salivary gland cells in which dHUWE1 was knocked down, including endoreplication and overall gland morphology. Moreover, knockdown of *dmP53* compensated for the loss of dHUWE1 in the adult eye, as well as in developing larval eye disc (Figure 5H–K). Likewise, overexpression of dMyc subsequently restored endoreplication and overall gland morphology, resulting in nuclei and glands that were larger than control. Expression of dMyc also restored adult eye morphology (Figure 5A–D’,G).

However, while co-eliminating *dm*P53 together with dHUWE1 suppressed ectopic activation of the JNK pathway reporter *puc^69^*::LacZ, co-expression of dMyc did not suppress the ectopic activation observed in cells in which dHUWE1 was co-targeted (Figure 5A”–D”). Moreover, expression of dMyc, but not dHUWE1 alone, resulted in the activation of the JNK pathway reporter (Supplementary Figure S5H–J). Remarkably, expression of UAS-dHUWE1 was not able to rescue the small and underdeveloped salivary glands the resulted from dMyc inactivation (Supplementary Figure S5K,L). These genetic interactions were also observed in the developing eye, where loss of dHUWE1 was compensated by expression of dMyc (Figure 5E–G). Thus, establishing one or more dHUWE1-regulated circuits during development.

## 4. Discussion

### 4.1. dHUWE1 Regulates Endoreplication

dHUWE1 is required for endoreplication of salivary gland cells. The inability of salivary gland cells to undergo endoreplication in the absence of dHUWE1 may stem from a failure to timely degrade cell-cycle regulators such as Cdc6, which is a known HUWE1 substrate in vertebrates, or other endocycle-regulating proteins involved in the E2F~CRL4 network, and the endoreplication oscillator [41,56,57]. In this context, it was recently reported that mammalian HUWE1 is the major enzyme catalyzing K6-linked ubiquitin chains. Among these K6-ubiquitinated substrates are numerous cell-cycle regulators that are subsequently targeted to the proteasome for degradation [9,58]. The potential existence of such endoreplication-specific regulators in *Drosophila* and their regulation by dHUWE1 awaits further studies.

### 4.2. dHUWE1 and the Regulation of the JNK Pathway

In this study we unveiled a role for dHUWE1 in the regulation of the JNK pathway. In contrast to the situation in *Drosophila*, HUWE1 enhances activation of the JNK pathway in mammalian cells. In cancer cells, the JNK pathway is inhibited by the Myc-interacting zinc finger transcription factor [59]. Miz-1 binds to TRAF2 and prevents its K63-polyubiquitination and subsequent pathway activation. Upon TNFα stimulation, HUWE1 ubiquitinates Miz-1, leading to its proteasomal degradation, alleviating Miz-1 inhibition, and enabling JNK pathway activation [59,60]. Interestingly, the *Drosophila* genome does not contain a clear ortholog of the Miz-1 protein, and this may explain the above difference in the regulatory function of HUWE1 within the JNK pathway.

Our genetic analysis suggests that HUWE1 impinges on the caspase-independent arm of the JNK pathway. However, the substrate(s) of dHUWE1 within the signaling cascade is yet to be identified. Epistatic studies suggest that potentially this substrate(s) may be found at the level of the TRAF6, TAB signaling complex. Indeed, HUWE1 was shown to catalyze branched ubiquitin chains on TRAF6 substrates during activation of NFκB, thus protecting these ubiquitinated proteins from degradation, and enabling recognition of TAB2, and subsequently amplifying NFκB signaling [10]. Similar ubiquitination may take place in salivary gland cells, together with a distinct E2 enzyme, but results in K6-linked ubiquitination that targets those signaling molecules to proteasomal degradation, inhibiting JNK pathway activation.

### 4.3. dHUWE1 and the Regulation of dMyc

In salivary gland cells, expression of dHUWE1 induces a slight increase in size of the nucleus. This fits well with a previous report linking dHUWE1 to a cell-growth/size control signaling pathways that includes translation initiation factors, as well as dMyc [42]. However, the molecular and genetic connection between the Myc protein family and HUWE1 is complex [5,19]. dHUWE1 was dispensable for the expression of bona-fide dMyc target genes and did not affect dMyc protein levels. Thus, unlike in vertebrates, dHUWE1 did not impact Myc stability or activity, yet the expression of dMyc restored endocycles in dHUWE1-targeted cells as well as in dHUWE1 loss-of-function eye phenotypes [61]. This ability of dMyc to compensate for the loss of dHUWE1 is evolutionarily conserved. Homeostatic defects observed in developing and mature B-cells observed in mouse B-cells conditionally deficient of HUWE1 were compensated by the expression of c-Myc in vivo [61]. Taken together with our genetic analysis, this suggests that a Myc-regulated pathway is situated downstream or parallel to dHUWE1 during development. In the context of the JNK pathway, however, dHUWE1 and dMyc exhibit reciprocating functions: While dHUWE1 inhibits JNK activation, dMyc enhances JNK activation in salivary gland cells. This is likely a context-specific function of dMyc, as dMyc was shown to antagonize the JNK pathway during retinal glial cell development [51].

### 4.4. Regulation of dHUWE1 by dmP53 in the Absence of Mcl1 and p14ARF

In vertebrates, HUWE1 regulates cell death by affecting several key death-inhibiting or promoting proteins. HUWE1 directly binds and ubiquitinates the anti-apoptotic protein Mcl1 on the mitochondria membrane, leading to Mcl1 degradation, thus enabling cell death [17]. In contrast, HUWE1 was also shown to ubiquitinate and degrade p53 [6,61], and was identified as a binding partner to ARF, inhibiting its activity (hence one of its names, ARF-BP1) [11,17]. Interestingly, the *Drosophila* genome lacks both *arf* and *mcl1*, but its genome codes for a conserved *dmP53*. Loss of *dmP53* suppressed all *dHUWE1* loss-of-function phenotypes, thus restoring endoreplication, preventing the ectopic activation of the JNK pathway, and restoring salivary gland and eye morphology. This suggests that in *Drosophila*, the anti-apoptotic activity of dHUWE1 is aimed directly at dmP53 or a dmP53-dependent process. Regardless of the exact mechanism involved, the observation that loss of *dHUWE1* resulted in apoptosis is consistent with results regarding the function of HUWE1 as observed in cancer cells. HUWE1 was identified as crucial for the tumorigenicity of cancer cells, and its genetic or pharmacologic targeting resulted in the collapse of tumor cells [5,62]. Our study therefore suggests that *Drosophil*a offers a trackable in vivo system for testing the regulation and interaction of HUWE1 with p53, as an evolutionarily conserved pathway of cell death.

## 5. Conclusions

We identified dHUWE1 as a key ubiquitin ligase that is essential for early developmental regulation of salivary gland morphogenesis. The salivary gland is instrumental in larval growth and undergoes massive expansion. It is, however, dispensable in the later stages of pupal development and degenerates during pupal stages. Our study suggests that dHUWE1 is required for extensive endoreplication of salivary gland cells during salivary gland development, and that it concomitantly safeguards the gland against premature activation of the JNK pathway that leads to its degeneration. Indeed, the mRNA level of dHUWE1 increases during larval development and sharply declines during pupal stages, enabling pathway activation. Moreover, our findings are relevant beyond salivary gland development and represent general functions of dHUWE1 that are highly relevant to other developing tissue, such as the eye, and at the organismal level. Thus, dHUWE1 serves as both a growth-promoting as well as a survival factor that is essential for development.

## Supplementary Materials

The following are available online at http://www.mdpi.com/2073-4409/7/10/151/s1, Supplementary Figure S1–S5.
