# Supplementary material for: Drosophila HUWE1 Ubiquitin Ligase Regulates Endoreplication and Antagonizes JNK Signaling During Salivary Gland Development
Source: Cells. 2018 Sep 26;7(10):151. doi: 10.3390/cells7100151 (PMC6210797; doi:10.3390/cells7100151)
Supplement: Supplementary file 1 [file cells-07-00151-s001.zip › cells-347889-SI.pdf]

Supplemental Figures 1-5

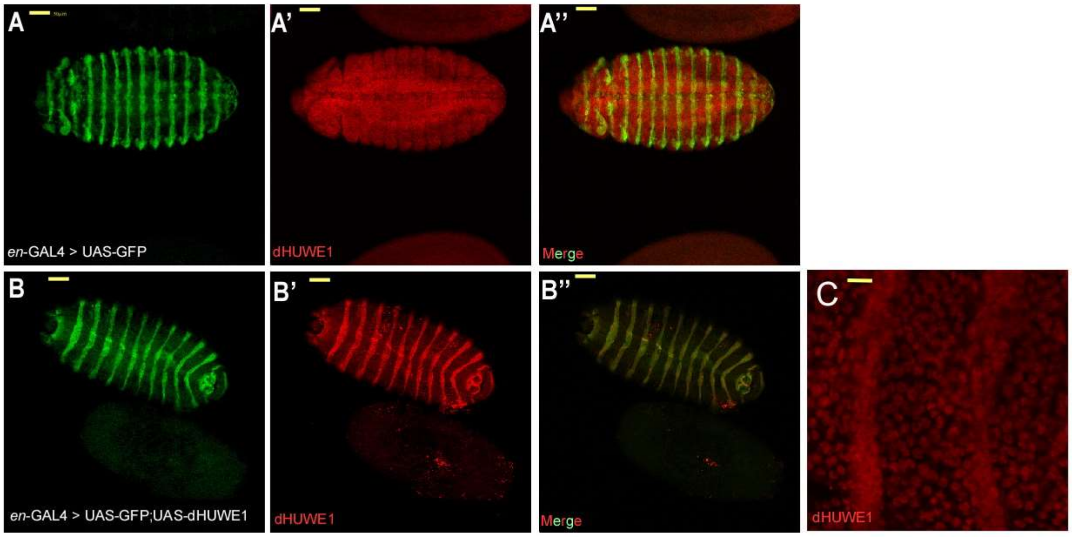

Figure S1. Expression pattern of endogenous and exogenous dHUWE1 . (A-C) Indirect immunofluorescence confocal microscopy images of stage 12 embryo expressing UAS-dHUWE1 and UAS-GFP under the control of *engrailed*-Gal4. Endogenous and ectopically expressed dHUWE1 is identified using polyclonal anti-dHUWE1. scale bar is 50μm (C) Magnification of a section of a stage-12 embryo [x120].

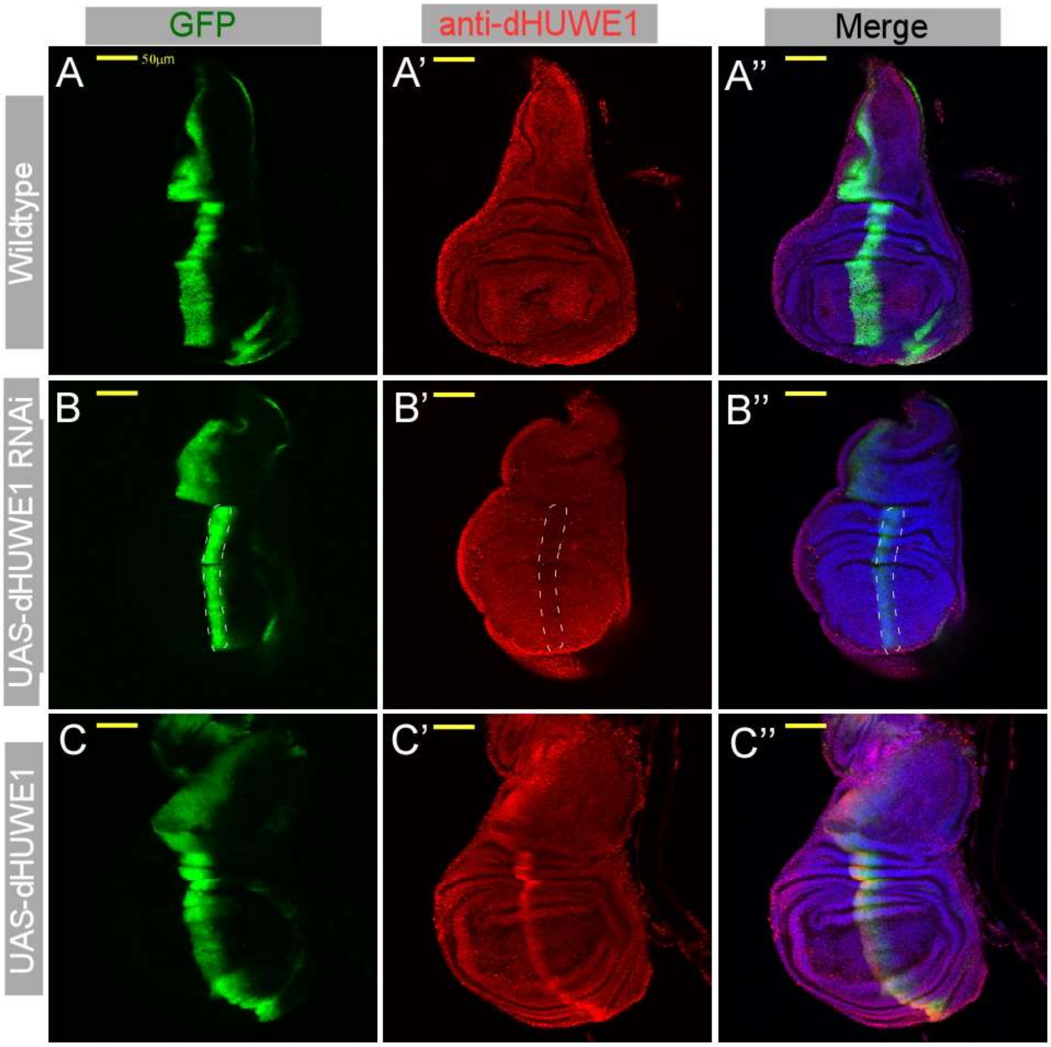

Figure S2. Expression and elimination of dHUWE1 protein in imaginal wing discs. (A-C) Expression of UAS-GFP, (A-A”) UAS-dHuwe1-RNAi, (B-B”) or UAS-dHUWE1 under the control of the *ptc*-Gal4 driver. GFP marks the region of *ptc*-GAL4 activity (dashed line). dHUWE1 is identified using anti-dHUWE1 (red). DRAQ5 marks DNA and scale bar is 50 μm.

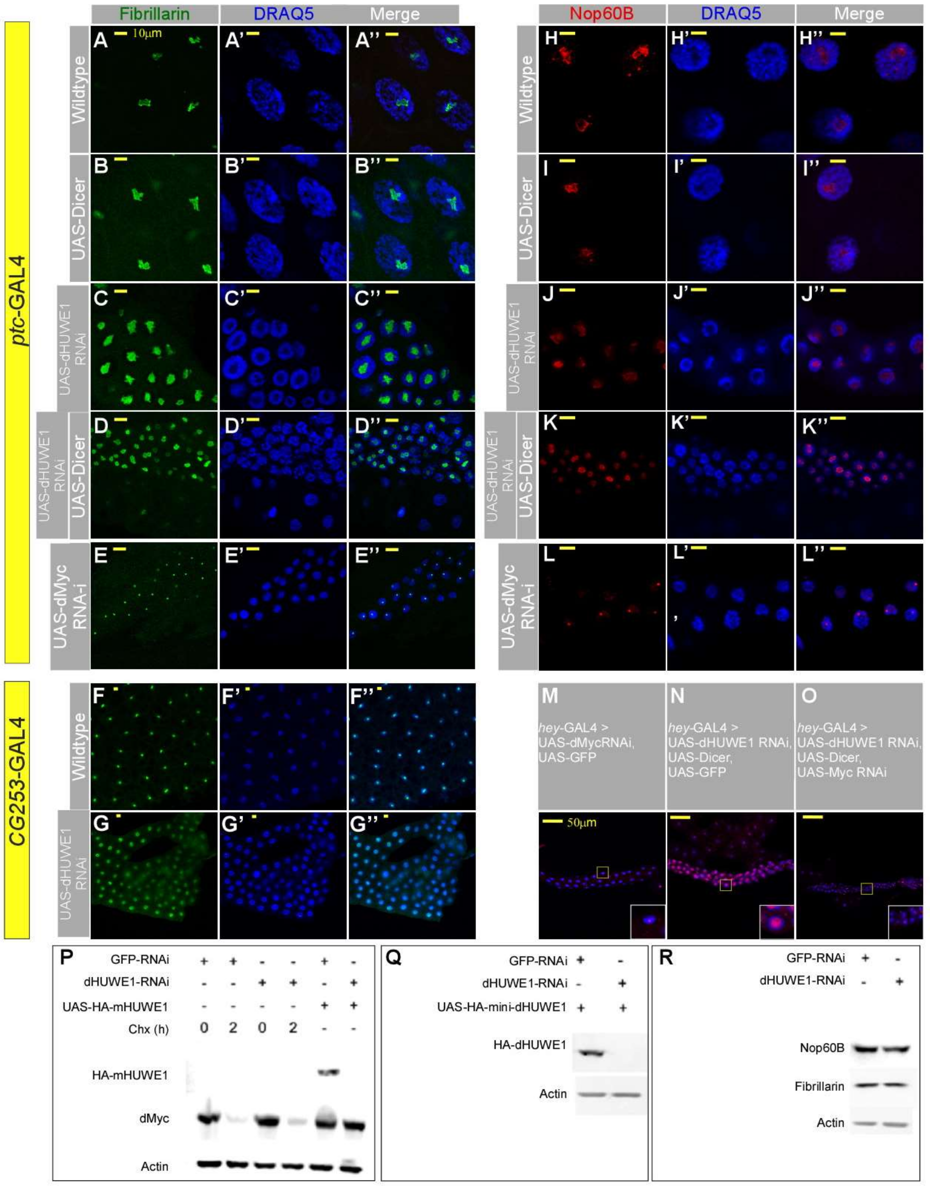

Figure S3. Expression of direct dMyc target genes is not affected by loss of dHUWE1. (A-J") Indirect immunofluorescence confocal microscopy images of salivary glands (A-E", H-O), and fat body tissue (F-G") derived from wild-type, or the indicated transgenic lines under the regulation of *hey*-Gal4 (A-E", H-O) and *CG235*-GAL4 (F-G"). (A-G") expression of Fibrillarin protein (green). (H-O) expression of Nop60B (red). DRAQ5 marks DNA and scale bar is 10 µm. **(P)** Protein level of endogenous dMyc in dynamic cyclohexamide chase experiment in S2 *Drosophila* cells transfected with the indicated RNAi. HA-dHUWE1 codes for a short C-terminal fragment of dHUWE1 that indicates the effectiveness of dHUWE1 knockdown, and actin serves as a loading control .**(Q, R)** Western blot analysis of the indicated protein extracts derived from S2 *Drosophila* cells transfected with either GFP-RNAi or dHUWE1-RNAi. Actin serves as a loading control.

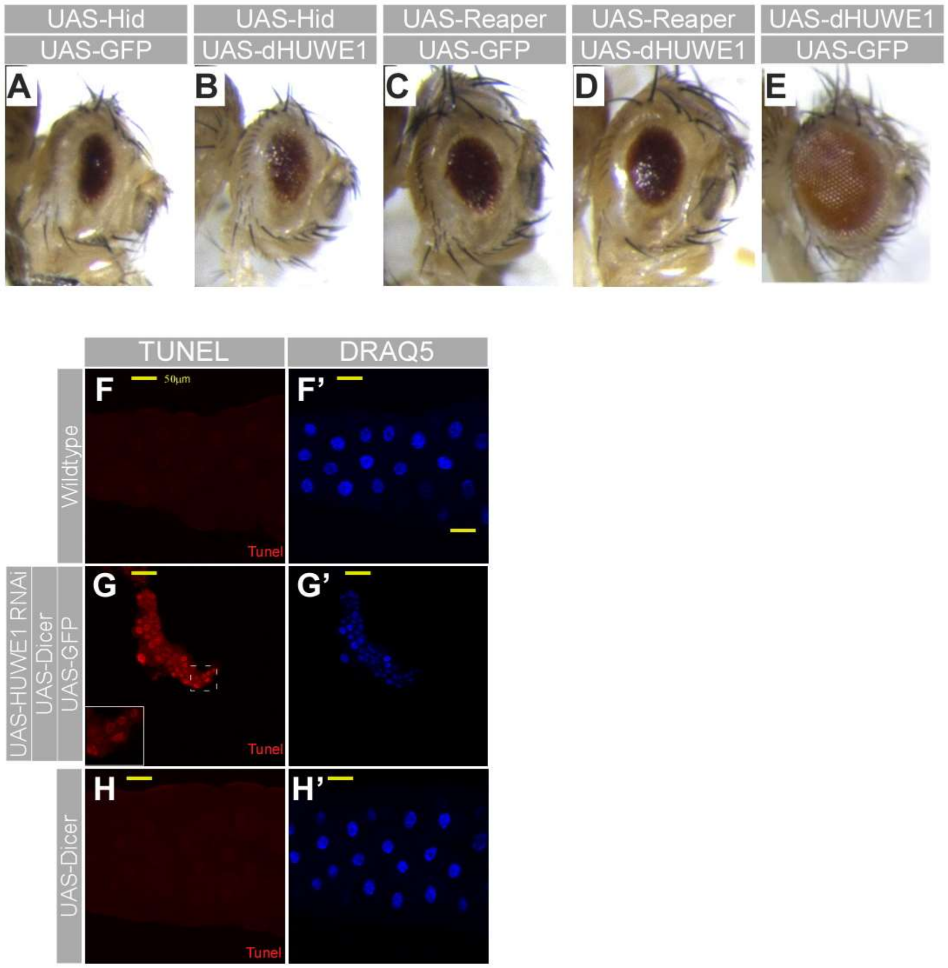

Figure S4. Expression of dHUWE1 fails to suppress cell death induced by the expression of Hid, or Reaper, and its loss initiates cell death (A-E) Expression of the indicated UAS-transgenic lines under the control of GMR>Gal4 driver. (A, C) Overexpression of either UAS-Hid (A) or UAS-Reaper (C), along with UAS-GFP control. Co-expression of UAS-dHUWE1 fails to suppress the small eye phenotype that results from overexpression of Hid (B) or Reaper (D). (F-H') Loss of dHUWE1 initiates cell death. TUNEL assay in salivary gland cells derived from the indicated transgenic lines. RNAi-mediated knockdown of dHUWE1 resulted in TUNEL positive cells. DRAQ5 marks DNA scale bar is 50µm.

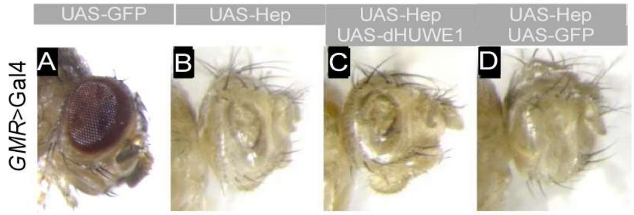

E

Genotype	n	% Small salivary gland
WT	40	0
dHUEW1^mut^;*ptc*-Gal4 > UAS-GFP	42	69
dHUEW1^mut^;*ptc*-Gal4 > UAS-Grnd RNAi 43454	39	18
dHUEW1^mut^;*ptc*-Gal4 > UAS-Traf 6 RNAi 110266	27	41
dHUEW1^mut^;*ptc*-Gal4 > UAS-Traf 6 RNAi 16125	52	10
dHUEW1^mut^;*ptc*-Gal4 > UAS-Tab 2 RNAi 100326	13	46

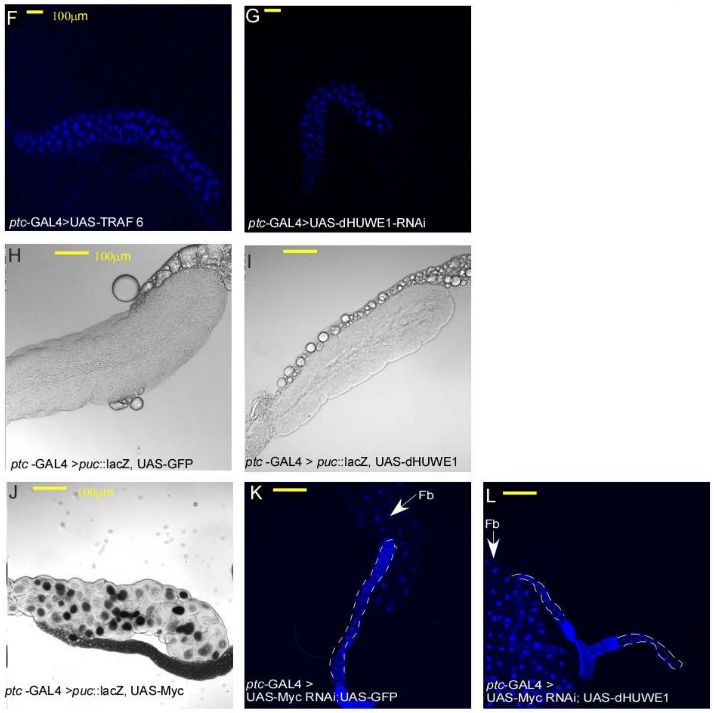

Figure S5: dHUWE1 genetically interacts with component the JNK pathway (A-D) dHUWE1 expression fails to suppress small eye phenotype induced by the expression of Hep. *ey*-Gal4 was used to express the indicated transgenes in the developing eyes. (E) Summary of epistatic analysis testing for genetic interaction between *dHUWE1^p^* heterozygous mutant, and the indicated transgenic RNAi lines under the control of *ptc*-GAL4 scoring for salivary gland morphology and size. "n" denotes number of salivary glands scored. (F, G) *ptc*-GAL dependent expression of UAS-TRAF6 (F) or RNAi-mediated knockdown of dHUWE1 (G) resulted in a small salivary gland and failure to fully endoreplicate. DAPI marks DNA and scale bar is 100µm. (H-J) Gross morphology and activation of JNK pathway as indicated by the expression of the JNK reporter transgene *puc*::LacZ (black circles) in salivary glands derived from the indicated transgenic lines. (K, L) Expression of either UAS-GFP (K), or UAS-dHUWE1 (L) failed to suppress the underdeveloped salivary gland that resulted from RNAi-dependent knockdown of dMyc.
